# Climate change and mental health: An overview

**DOI:** 10.1192/j.eurpsy.2021.50

**Published:** 2021-08-13

**Authors:** A. Erfurth, G. Sachs

**Affiliations:** 1 1st Department Of Psychiatry And Psychotherapeutic Medicine, Klinik Hietzing, Vienna, Austria; 2 Department Of Psychiatry And Psychotherapy, Medical University of Vienna, Vienna, Austria

**Keywords:** air pollution, climate change, mental health, Urbanicity

## Abstract

According to the UN Environment Programme “climate change is one of the most pervasive and threatening issues of our time”. “In many places, temperature changes and sea-level rise are already putting ecosystems under stress and affecting human well-being” (1). The presentation wants to give an overview on how climate change can affect mental health. A search was performed on PubMed for the combination of “climate change” and “mental health”. 281 publications were identified, the first being from 2007 (the only one in that year). In 2020, until Dec 22^nd^, 76 publications were found. The somehow prophetic 2007 publication reviews “natural disasters, climate change and mental health considerations for rural Australia” (2) and pinpoints central aspects of today’s debate, namely anxiety and depression, vulnerability and resilience. In addition to problems of rural areas (2), the impact of urbanicity (3) will be discussed as well as the role of air pollution on psychiatric disorders (4). (1) UN Environment Programme. https://www.unenvironment.org/explore-topics/climate-change/about-climate-change Dec 22^nd^, 2020. (2) Morissey SA, Reser JP. Aust J Rural Health. 2007 Apr;15(2):120-5. doi: 10.1111/j.1440-1584.2007.00865.x. (3) Krabbendam L et al. Psychol Med. 2020 Mar 11:1-12. doi: 10.1017/S0033291720000355. (4) Kim SY et al. Sci Total Environ. 2020 Dec 8;757:143960. doi: 10.1016/j.scitotenv.2020.143960.

**Disclosure:**

No significant relationships.

